# The First Registered Type 0 Spinal Muscular Atrophy Patient in Latvia: Call for Change in Prenatal Diagnostic Procedures

**DOI:** 10.1155/2023/3480298

**Published:** 2023-06-01

**Authors:** Tīna Luīze Čupāne, Mikus Dīriks, Gita Tauriņa, Liene Korņejeva, Linda Gailīte, Ieva Mālniece, Madara Auzenbaha

**Affiliations:** ^1^University of Latvia, Faculty of Medicine, Riga, Latvia; ^2^European Reference Network EURO-NMD, Paris, France; ^3^Children's Clinical University Hospital (CCUH), Department of Neurology and Neurosurgery, Riga, Latvia; ^4^Children's Clinical University Hospital (CCUH), Medical Genetics and Prenatal Diagnostic Clinic, Riga, Latvia; ^5^The Riga Maternity Hospital, Riga, Latvia; ^6^Riga Stradins University, Scientific Laboratory of Molecular Genetics, Riga, Latvia

## Abstract

This case report presents the first registered patient in Latvia with type 0 spinal muscular atrophy (SMA). During the first-trimester ultrasonography of the unborn patient, an increased thickness of the nuchal fold was detected. The mother reported decreased foetal movements during the pregnancy. After the boy was born, his general condition was extremely severe. The clinical signs indicated a suspected neuromuscular disorder. A precise diagnosis, type 0 SMA, was determined 7 days after birth through a newborn pilot-screening for SMA, which was conducted for all newborns whose parents consented to participate. The condition of the infant deteriorated. He had severe respiratory distress followed by multiple events leading to his death. Currently, there are only a few published case reports detailing an increased nuchal translucency (NT) measurement in association with a diagnosis of SMA in the foetus. However, an increased NT measurement is a clinically relevant sign as it can be related to genetic syndromes, foetal malformations, disruptions, and dysplasias. Since there is no cure for infants with type 0 SMA at present, it is crucial to be able to detect this disease prenatally in order to provide the best possible care for the patient and parents. This includes the provision of palliative care for the patient, among other measures. This case report highlights the prenatal signs and symptoms in relation to type 0 SMA.

## 1. Introduction

Spinal muscular atrophy 5q (SMA5q) is a genetic disease characterised by the degeneration of anterior horn cells in the spinal cord and motor nuclei in the lower brainstem [1]. The most common cause is the deletion of exon 7 in the survival motor neuron 1 (*SMN1*) gene. The severity of the disease correlates with the number of copies of the survival motor neuron 2 (*SMN2*) gene [2]. The SMN protein is synthesized by two genes: *SMN1* and *SMN2*. In each cell, there are typically two copies of the *SMN1* gene and one or two copies of the *SMN2* gene. The number of *SMN2* gene copies can vary, with some individuals having up to eight copies. Although the *SMN2* and *SMN1* genes produce proteins that are identical, only a small fraction of functional protein, about 10 to 15 percent, is synthesized by the *SMN2* gene, with the majority being produced by the *SMN1* gene. Therefore, the number of *SMN2* gene copies a person has can significantly impact the amount of SMN protein they can generate [2, 3].

According to available data, the incidence of the disease in Latvia is 1/9,091 [4]. As of 2021, the population of Latvia was approximately 1.89 million people [5], with 17,420 live births recorded [6]. Newborn screening has been implemented in Latvia since April 2023 [7], following a pilot study that offered the opportunity to participate in an expanded screening program starting on December 1, 2020 [8]. The global incidence of spinal muscular atrophy is about 1 in 10,000 live births [9]. The carrier frequency worldwide is around 1 in 35 [10, 11]. Patients in Latvia that are diagnosed with spinal muscular atrophy have two treatment options: risdiplam and nusinersen. Nusinersen received European approval in June 2017 [12] and became accessible in Latvia by July 2019. More recently, risdiplam received European approval in February 2021 [13] and became accessible in Latvia by February 2022.

The disease is classified into five types depending on the age of onset and the clinical course [14]. The most severe type is SMA type 0, and the least severe is SMA type 4 (Table 1). SMA type 0 manifests prenatally. Mothers of affected children can experience a decrease or even a loss of foetal movements during pregnancy [16]. In addition, foetuses may present with intrauterine growth retardation, pulmonary hypoplasia, and skeletal abnormalities. Newborns with SMA type 0 have a severe muscle weakness and hypotonia at birth. They often present with areflexia, congenital heart defects, and facial diplegia [17]. Affected infants are not able to achieve any motor function milestones. Most babies die by one month of age due to respiratory failure. Patients usually present with one copy of the *SMN2* gene [18].

### 1.1. Case Presentation

This case report presents the first registered patient in Latvia with type 0 SMA. The index case was a male infant from nonconsanguineous parents. The mother was a 33-year-old Caucasian female. She did not smoke and had no health issues. The father was a 35-year-old Caucasian male with no chronic illnesses. There was no positive family history of SMA; however, both parents were carriers of a mutated *SMN1* gene. The index patient was their second baby. The previous pregnancy was uneventful, and a healthy child was born. Thus far, no SMA5q symptoms have been reported.

The mother's pregnancy with the proband was considered high risk due to her age. Consequently, several ultrasonographic parameters (thickness of foetal nuchal translucency (NT), nasal bone length, tricuspid valve and ductus venosus dopplerometry, and foetal heart rate) were assessed, as were maternal serum biochemical parameters. Chorionic villus sampling was performed as a precautionary measure due to the increased risk of aneuploidy, changes observed in ultrasound readings, and maternal age.

During the first-trimester ultrasonography, an increased thickness of the nuchal fold (7.9 mm; <2.5 mm or <95^th^ percentile, places the pregnancy at a low risk; ≥6.5 mm, places the pregnancy at a high risk [19]) was detected as well as a cystic hygroma. No heart defects were detected. Chorionic villus sampling excluded common chromosomal aneuploidies by quantitative fluorescent PCR. Furthermore, chromosomal microarray analysis did not show any pathological variations. The mother reported reduced foetal movements throughout the pregnancy, especially during the third trimester. Despite a normal chromosomal microarray analysis result, a high index of suspicion for genetic pathology was maintained in view of abnormal ultrasonographic findings. However, extensive panel genetic testing using next-generation sequencing or exome sequencing was not performed as it is not a routine prenatal test in Latvia.

The patient was born at 36^+1^ weeks of gestational age. Due to pelvic presentation, followed by an external cephalic version and bradycardia, an acute caesarean section was performed. The weight of the neonate was 2,950 grams (17^th^ percentile) and the length was 50 cm (52^nd^ percentile). The head circumference was 35 cm (66^th^ percentile).

After the birth, the general condition of the infant was extremely severe. The patient had severe asphyxia during labour. Primary resuscitation was initiated, comprising tactile stimulation, upper airway suctioning, and ventilation. The patient had severe respiratory distress, no active movements, atonia, and areflexia, and his skin was cyanotic. The Apgar scores were 1/1/2/3. To provide ventilatory support, intubation was required in the first minute after birth; however, the patient's condition deteriorated, and so mechanical ventilation was implemented. The patient experienced a femur fracture and newborn hypoglycemia and was later diagnosed with an atrial septal defect.

To identify the cause of the infant's severe condition, TORCH infections were checked for and excluded, a head MRI was performed, and a geneticist was consulted. The head MRI results reported multiple ischaemic foci in the frontal and parietal lobes and in the basal ganglia. Based on the patient's clinical signs and symptoms, the geneticist suspected SMA. The parents consented to newborn screening for SMA5q and 7 days after birth, a homozygous deletion of exon 7 in the *SMN1* gene was reported. Further genetic analysis as part of the Latvian newborn screening protocol detected 0 copies of the *SMN1* gene and 1 copy of the *SMN2* gene. A diagnosis of type 0 SMA was confirmed.

Ten days after their son's birth, a multidisciplinary team informed the parents about his type 0 SMA diagnosis. They were informed about the limited treatment options and poor prognosis. Following a full discussion, the parents decided against invasive mechanical ventilation, and the infant was extubated. Rapid desaturation, bradycardia, and cardiac arrest followed and the patient died.

The concluding diagnoses of the neonate were as follows: type 0 SMA, severe asphyxia during labour, femur fracture, atrial septal defect, and newborn hypoglycemia.

## 2. Discussion

We report here the case of a newborn with type 0 SMA. At the first-trimester ultrasonography, an increased NT thickness was detected. After birth, the patient had severe respiratory distress followed by multiple events leading to a fatal outcome.

Currently, the identification of SMA-affected infants prior to the presentation of clinical symptoms is accomplished by newborn screening. However, the severity of this disorder highlights the importance of prenatal detection.

NT thickness evaluation is associated with multiple foetal malformations, genetic syndromes, intrauterine death risk, congenital heart defects, and a high risk of miscarriage. Most of the structural anomalies are undetectable prior to childbirth [20]. There are only a few published case reports of an increased NT measurement in association with a diagnosis of SMA type 0 in the foetus [21].

The study by Parra et al. explored the correlation between increased NT in SMA foetuses and the severity of the disease. The findings suggested that a thickened NT is an infrequent occurrence in most foetuses that are affected by SMA, but it may indicate the relevance of *SMN2* copy number in the development of congenital heart defects and elevated NT values. The research highlighted the significance of early echocardiography to monitor at-risk foetuses and detect potential complications [22]. In some cases, it may present with multiple health issues, as evidenced by our case involving a newborn diagnosed with SMA type 0. The index case also had severe asphyxia during labour, femur fracture, atrial septal defect, and newborn hypoglycemia, illustrating the complexity and severity of type 0 SMA and its associated complications.

It is well established that an increased NT thickness is predictive value for an adverse pregnancy outcome, even if conventional karyotyping is normal. The risk of foetal malformations is proportional to the NT thickness [20]. Specifically, if the enlargement is between 3.5 and 4.4 mm, the risk of a foetal chromosome abnormality is 21%, rising to 33% if it is 5 mm, 50% if it is 6 mm, and 65% if it is greater than 6.5 mm [23]. However, multiple studies have shown no association between an increased NT thickness and the diagnosis of SMA, suggesting that SMA-affected foetuses have normal NT thickness values. For instance, a study by Zadeh et al. investigated 12 SMA-affected infants with confirmed NT thickness results during pregnancy. All the foetuses had normal NT thickness values ranging from 0.7 to 2.4 mm, implying the SMA is not associated with an enlarged NT [24]. Barone and Bianca examining 29 women proposed that foetal genetic testing of the *SMN1* gene on the basis of increased NT thickness is not indicated in couples with no previous history of SMA [25]. Nevertheless, the findings of Parra et al. support the idea that *SMN2* gene copy number in SMA foetuses is relevant for the development of congenital heart defects and increased NT thickness values [22].

A broad range of options is needed to help diagnose SMA prenatally. The gold standard for SMA diagnosis is multiplex ligation probe analysis [26], a targeted test. However, as an increased NT thickness is not currently considered a sign of SMA, this would not have been employed in the case described here. If the signs are not clear, then prenatally the best next-generation sequencing method is exome sequencing; however, it requires specific bioinformatic analysis [27], as *SMN1* and *SMN2* are genes with high homology. If a neuromuscular disorder is suspected, then targeted neuromuscular disease panels can be used, as described by Zhao et al. and Tan et al. [28, 29].

The mother's observation of decreased foetal movements served as an additional indication of potential pathology for the unborn child. Nonetheless, research suggests that only in rare cases are reduced foetal movements associated with foetal neuromuscular disorders. In our case, the reduced movements suggested that the symptoms of a neuromuscular disorder were already present before birth. Studies show that in cases of SMA type 0, reduced foetal movements are typically experienced between 30 and 36 weeks of gestation and are associated with a markedly restricted lifespan [30].

The findings reported by Grotto et al. regarding the clinical manifestations of SMA type 0 are consistent with those observed in the patient presented in our report [16]. The importance of early diagnosis and management of the disease is enhanced. As noted by Andrea et al., the lack of a specific and proven treatment for SMA type 0 underscores the need for further research in this area. Well-designed studies are necessary to assess potential treatments. In the meantime, healthcare providers should focus on providing appropriate palliative care and genetic counseling to affected families. Early diagnosis, preferably even before birth, can aid in the provision of optimal care to patients with SMA type 0 [31].

The reported cases of type 0 SMA have been ventilator dependent and have ultimately passed away in the neonatal period after ventilation support was withdrawn, usually at the request of parents. Our case was no different. The infant was ventilator-dependent and passed away after ventilation support was withdrawn at the request of the parents [16, 31, 32].

In rare cases, type 0 SMA can be accompanied by additional features, known as SMA plus. A case report by Gathwala et. al shed light on a case of SMA type 0 in combination with a Dandy–Walker variant, which was confirmed through molecular genetic analysis that revealed the homozygous deletion of exon 7 of the *SMN1* gene. After the patient was born, a CT scan was conducted. It showed partial vermian hypoplasia and partial obstruction in the fourth ventricle. There was no enlargement observed in the posterior fossa. These results were consistent with a diagnosis of a Dandy–Walker variant. While SMA plus is not uncommon and various additional features have been described, the combination of SMA type 0 with the Dandy–Walker variant is particularly rare. These case studies highlight the need for increased awareness and understanding of the complex nature of SMA and its associated conditions [32].

In the case described here, more comprehensive diagnostic procedures should have been performed to try and identify the cause of the abnormal NT result prenatally. This information would have given the parents the option of terminating the pregnancy. In addition, carrying out a more thorough evaluation to determine the cause of decreased foetal movements, along with the abnormal NT result, would have led to improved patient care and enhanced support for the parents. Such care could have included palliative care for the baby and enhanced psychological support for the parents. However, a major consideration in this case was the financial support provided by the parents' health insurance, which did not allow for extensive diagnostic procedures.

Innovative treatments have been developed for patients with SMA. However, the treatment options for type 0 SMA patients are limited [33]. These patients require respiratory and feeding support, which typically is not enough to keep the patients alive. A recent case report from Matesanz et al. described the clinical course of a patient with type 0 SMA who was treated with nusinersen and onasemnogene abeparvovec. Although the infant had modest motor improvements, she also had continued systemic complications from her SMA, thus highlighting the challenges of treating patients with more severe phenotypes of SMA. Nevertheless, the possibility that the genetic treatment had a positive effect compared to the original course of the disease cannot be denied. Similar to our case, this patient had multiple medical conditions that were related to their underlying issue, including cardiac abnormalities such as atrial and ventricular septal defects. Studies propose that individuals with a lower copy number of *SMN2* and a more severe phenotype of spinal muscular atrophy are more likely to have structural heart malformations [34].

Tiberi et al.‘s case report presented the case of a type 0 SMA patient with 1 copy of the *SMN2* gene. At the age of 13 days, the infant received treatment with nusinersen. Although he showed mild motor improvement, he required a tracheostomy at the age of 4 months. An increasing cardiac and autonomic dysfunction followed, and he died at the age of 5 months [25]. Therefore, the available data suggest that despite the development of innovative treatments for SMA, they are unlikely to produce a significant positive effect in type 0 patients.

According to a study, the reported incidence of SMA in Latvia is 1 in 9,091 [4]. The first results of a pilot-screening involved an examination of 10,411 patients, resulting in the identification of SMA 5q-positive cases at a frequency of 1 in 5,205 [35]. However, the duration of the screening period was insufficient to firmly establish this incidence rate. Between December 2021 and April 2023, a pilot screening for SMA was conducted on 26,421 newborns in Latvia. It gave a valuable insight on this condition. Previously unpublished data revealed 3 confirmed cases of SMA, suggesting a slightly higher incidence rate than previously reported. The new findings suggest that the occurrence of SMA in Latvia is approximately 1 out of every 8,807 individuals. As a proactive measure, starting in April 2023, SMA has been integrated into the newborn screening program in Latvia. This holds promising implications and enhances the likelihood of early detection and administration of presymptomatic treatment for newborns affected by spinal muscular atrophy.

Despite the fact that the outcome expectancy of patients with type 0 SMA is pessimistic and the disease leads to early mortality, newborn screening for SMA5q can significantly improve the life expectancy and quality of patients with other types of SMA. There is evidence that presymptomatic treatment initiated as a result of newborn screening improves the outcome of children with genetically proven SMA. Including newborn screening for SMA is vital in order to initiate treatment presymptomatically and obtain the best outcome for patients [36]. In our case, the parents' other child has not been tested for SMA yet. The child displays no observable indications of a neuromuscular disorder and has not undergone evaluation by a geneticist. However, it would be prudent for the child to seek consultation with a geneticist and a neurologist if any symptoms appear.

## 3. Conclusion

Since there are no treatment options for infants with type 0 SMA at present, it is vital to be able to detect this disease prenatally in order to provide the best possible care for the patient and parents including the provision of palliative care for the patient, if needed. An increased NT is a clinically relevant sign that can be related to multiple foetal malformations, genetic syndromes, intrauterine death risk, congenital heart defects, and high risk of miscarriage. However, at present, there are only limited data supporting an association between NT enlargement and SMA.

Employment of extensive panel genetic testing using next-generation sequencing would be beneficial for the prenatal diagnosis of type 0 SMA cases. Moreover, sequencing of foetal exomes covering single nucleotide variations and copy number variations (also *SMN1*) offers a broader diagnostic capacity for pregnancies with unexpected foetal anomalies, thus improving both diagnostic timing and counseling for parents. Early recognition of the disease would give parents time to come to terms with the situation as well as provide the option of pregnancy termination.

## Figures and Tables

**Table 1:** SMA5q types [10, 14, 15].

SMA types	SMN2 copies	Ages of onset	Motor abilities	Prognoses
Type 0	1	Before birth	Very severe hypotonia. The patient cannot reach any motor function milestones.	The baby presents with respiratory insufficiency at birth. Death within a few weeks.
Type 1	2	<6 months	Severe hypotonia. The patient cannot sit or roll.	The symptoms progress rapidly. Most infants die before the age of 2 years.
Type 2	3	6–18 months	Proximal muscle weakness. The patient cannot walk independently.	Patients can have a shortened life expectancy. Frequently live into adulthood.
Type 3	3-4	>18 months	The patient can lose the ability to walk.	Usually life expectancy is not affected.
Type 4	4–8	Adulthood	The patient has a slight motor function impairment.	Life expectancy is not affected.

## Data Availability

The data generated during and/or analyzed during the current study are available from the corresponding author upon reasonable request. Access to the data is subject to any ethical or legal restrictions imposed by the relevant research institutions or governing bodies. To obtain the data, interested parties can contact the corresponding author, Tina Luize Cupane, via e-mail at tlcupane@gmail.com. The corresponding author will provide the data promptly and ensure compliance with any applicable data protection and privacy regulations.
